# Ethenesulfonyl fluoride derivatives as telomerase inhibitors: structure-based design, SAR, and anticancer evaluation *in vitro*

**DOI:** 10.1080/14756366.2018.1484735

**Published:** 2018-08-24

**Authors:** Xing Chen, Gao-Feng Zha, Jie Quan Wang, Xin-Hua Liu

**Affiliations:** a School of Pharmacy, Anhui Province Key Laboratory of Major Autoimmune Diseases, Anhui Institute of Innovative Drugs, Anhui Medical University, Hefei, P. R. China;; b School of Chemistry, Chemical Engineering and Life Science, Wuhan University of Technology, Wuhan, P. R. China;; c School of Material Science Chemical Engineering, ChuZhou University, ChuZhou, P. R. China

**Keywords:** Ethenesulfonyl fluoride, selective anticancer activity, telomerase, inhibitor

## Abstract

Based on our previous docking model, in order to carry out more rational drug design, totally 82 vinyl sulfonyl fluorides, including some 2-(hetero)arylethenesulfonyl fluorides and 1,3-dienylsulfonyl fluorides derivatives as potential human telomerase inhibitors were designed and synthesised. The *in vitro* anticancer activity assay showed that compound **57** (1*E*,3*E*)-4-(4-((*E*)-2-(fluorosulfonyl)vinyl)phenyl)buta-1,3-diene-1-sulfonyl fluoride exhibited high activity against A375 and MDA-MB-231 cell lines with IC_50_ 1.58 and 3.22 µM, but it manifested obvious un-toxic effect against GES-1 and L-02 with IC_50_ with IC_50_ values less than 2.00 mM. By the modified TRAP assay, some compounds including **57** exhibited potent inhibitory activities against telomerase with IC_50_ values of 0.71–0.97 µM.

## 1. Introduction

Telomerase plays an important role in chromosomal integrity. About 80–90% of various cancer cells have detectable telomerase activity, so, it is considered as a potential anticancer target^1–4^. In the past decade, based on searching for telomerase inhibitors, different approaches have been designed containing G-quadruplex stabilising inhibitors^5^^,^^6^, 2′-O-MeRNA oligonucleotides and peptide nucleic acids targeting telomerase RNA^7^, ligands targeting telomeric DNA^8^. But, no compounds that inhibit telomerase activity have reached clinical trials so far^9^.

Among the whole telomerase, human telomerase reverse transcriptase (TERT) is a key component of telomerase. The expression of TERT is a rate-limiting factor for telomerase activity; also, most human somatic cells do not show detectable telomerase activity due to the lack of TERT. Therefore, TERT is an important target for the drug discovery^10–13^. Some hTERT inhibitors with good anticancer activity, including BIBR1532^14^ and isothiazolone derivatives have been discovered^15–19^. However, most of them exhibited potential toxicities against somatic cells, there are no inhibitors targeting hTERT have been approved so far. Therefore, the design of potent hTERT inhibitors with high selectivity against cancer cells and somatic cells is a very important and immediate need.

**Figure 1.**
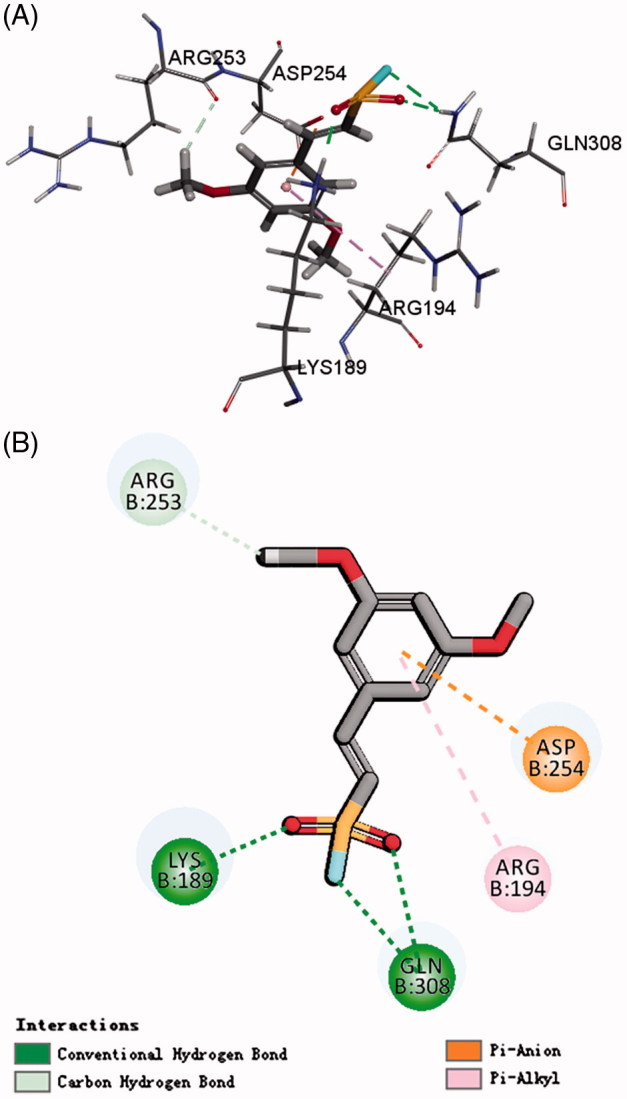
Rational design of target compound. (A) 3D picture of binding was depicted; (B) 2D picture of binding was depicted.

As we know, chemical environment of the warhead plays a key role in inhibitor design since it enables the tuning of its reactivity and thereby increasing its selectivity and stability^20^^,^^21^. Among them, vinyl sulfone, which is a dominant electrophilic trap containing unsaturated sulfone Michael acceptor, which could inhibit both the proteasome and cysteine proteases and high selectivity through manipulation of the peptidic portion^22^. Based on the structure of protein TERT using a LigandFit module, the novel docking model was found. The results showed that LYS 189, GLN 308, and ASP 254 were the key residues for telomerase activity. Focusing on these residues and the volume of the active site of hTERT, the fragment of ethenesulfonyl fluoride could be well aligned with the active site. We, therefore, designed some of the drug-like scaffolds which incorporate the moiety of ethenesulfonyl fluoride derivatives and tested their inhibition activity of telomerase (Figure 1).

## 2. Experimental

### 2.1. Cell proliferation assays

The antiproliferative activities evaluation was conducted by using a modified procedure as described in the literature^23^. Briefly, target tumour cells were grown to log phase in RPMI 1640 medium supplemented with 10% fetal bovine serum. After diluting to 3 × 10^4^ cells mL^−1^ with the complete medium, 100 µL of the obtained cell suspension was added to each well of 96-well culture plates. The subsequent incubation was performed at 37 °C, 5% CO_2_ atmosphere for 24 h before subjecting to antiproliferation assessment. The tested samples at pre-set concentrations were added to six wells with Doxorubicin (AMD) co-assayed as a positive reference. After 48 h of exposure period, 25 µL of PBS containing 2.5 mg·mL^−1^ of MTT was added to each well. After 4 h, the medium was replaced by 150 µL DMSO to dissolve the purple formazan crystals produced. The absorbance at 570 nm of each well was measured using an ELISA plate reader. The data represented the mean of three experiments in triplicate and were expressed as means ± SD using Student’s test. The IC_50_ value was defined as the concentration at which 50% of the cells could survive.

### 2.2. Telomerase activity assays

Thirty-six compounds were tested in a search for inhibitors of telomerase activity using the TRAP-PCR-ELISA assay. In detail, the A375 cells were firstly maintained in RPMI 1640 buffer (Hyclone, Miami, FL), supplemented with 10% fetal bovineserum (GIBCO, New York, NY), streptomycin (0.1 mg/mL) and penicillin (100 IU/mL) at 37 °C in a humidified atmosphere containing 5% CO_2_. After trypsinisation, 5 × 10^4^ cultured cells in logarithmic growth were seeded into T25 flasks (Corning, New York, NY) and cultured to allow to adherence. The cells were then incubated with Staurosporine (Santa Cruz, Santa Cruz,CA) and the drugs with a series of concentration as 60, 20, 6.67, 2.22, 0.75, 0.25 and 0.082 g/mL, respectively. After 24 h treatment, the cells were harvested by cell scraper orderly followed by washing once with PBS. The cells were lysed in 150 µL RIPA cell lysis buffer (Santa Cruz, Santa Cruz, CA), and incubated on ice for 30 min. The cellular supernatants were obtained via centrifugation at 12,000*g* for 20 min at 4 °C and stored at –80 °C. The TRAP-PCR-ELISA assay was performed using a telomerase detection kit (Roche, Basel, Switzerland) according to the manufacturer’s protocol. In brief, 2 µL of cell extracts were mixed with 48 µL TRAP reaction mixtures. TRAP primers and Taq polymerase are incubated at 25 °C for 30 min. PCR was then initiated at 94 °C, 120 s for predenaturation and performed using 35 cycles each consisting of 94 °C for 30 s, 50 °C for 30 s, 72 °C for 90 s. Then 20 µL of PCR products were hybridised to a digoxigenin (DIG)-labelled telomeric repeat specific detection probe. And the PCR products were immobilised *via* the biotin-labelled primer to a streptavidin-coated microtiter plate subsequently. The immobilised DNA fragments were detected with a peroxidase-conjugated anti-DIG antibody and visualised following the addition of the stop regent. The microtitre plate was assessed on TECAN Infinite M200 microplate reader (Mannedorf, Switzerland) at a wavelength of 490 nm, and the final value were presented as mean ± SD^23^.

### 2.3. Molecular modelling

The Discovery Studio 2017 was used (Accelrys Software Inc., San Diego, CA). Crystal structure of telomerase TERT (PDB: 3DU6) was used as a template. The active site was defined and sphere of 5 Å was generated around the active site pocket, with the active site pocket of BSAI model using C-DOCKER. The structure of protein, substrate were subjected to energy minimisation using CHARMm forcefield as implemented in DS 2017.

### 2.4. Statistical analysis

All results are expressed as Mean ± SE. Statistical significance was determined either by the Student’s *t*-test for comparison between means or one-way analysis of variance with a *post-hoc* Dunnett’s test.

## 3. Results and discussion

### 3.1. Chemistry

**Figure 2.**
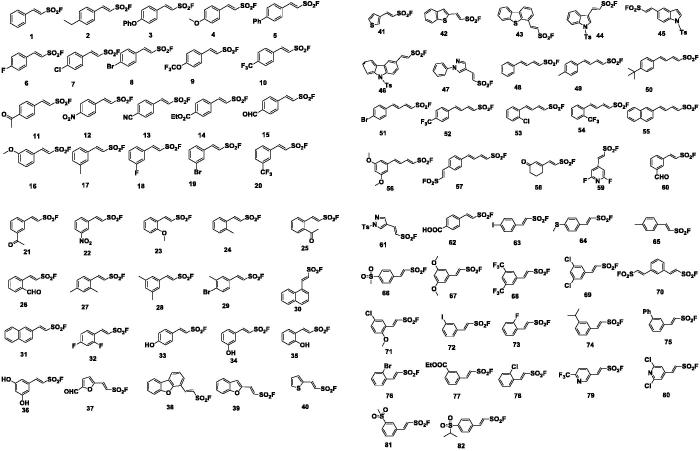
Structures of compounds 1–82.

In our previous study, 82 structurally diverse vinyl sulfonyl fluorides, including some 2-(hetero)arylethenesulfonyl fluorides and 1,3-dienylsulfonyl fluorides, were synthesised on a decent scale using a general method. Our results of molecular construction revealed that Heck-type reaction had a broad scope for aryl iodides comprising various types of ethenesulfonyl fluoride derivatives. Under mild reaction conditions used simple operations, catalytic amounts of Pd(OAc)_2_ and stoichiometric amounts of AgTFA affected the transformations of aryl iodides to the corresponding vinyl sulfonyl fluorides with good yields (Figure 2)^24^.

### 3.2. Anticancer activity

Compounds **1**–**82** were evaluated for their antiproliferative activities against human melanoma cell A375, human breast cancer cell MDA-MB-231, human hepatoma cell SMMC-7721, human gastric cancer cells SGC-7901 and MGC-803 cell lines. The cells were allowed to proliferate in the presence of tested material for 48 h, and the results were reported with IC_50_ values. The IC_50_ values of all tested compounds against five cell lines are summarised in Table 1. From the structure-activity relationships presented, in general, it can be concluded that almost all compounds displayed poor activity against SGC-7901 and MGC-803 cell lines (except for compound **70**). Some of the compounds showed moderate activity to MDA-MB-231 cell (such as compounds **1, 5, 6, 11, 12, 18, 22, 35, 69, 70**, **71** the IC_50_s values are between 4.22 and 8.67 µM), among them, compounds **14**, **15**, **20** and **57** exhibited high activity against MDA-MB-231 with IC_50_ values at 3.60, 3.64, 3.18 and 3.22 µM, respectively. Compared with MDA-MB-231, the title compound had a certain activity for SMMC-7721 cell, but it is generally weaker than that of MDA-MB-231. One of them, compound **30** is the most potential compound with IC_50_ value of 2.29 µM, which is comparable with the positive control AMD.

**Table 1.** Antiproliferative activity of compounds **1**–**82** against A375, MDA-MB-231, SMMC-7721, SGC-7901 and MGC-803 cell lines.^a^

Compound	IC_50_ (μM)				
	A375	MDA-MB-231	SMMC-7721	SGC-7901	MGC-803
**1**	5.21 ± 0.39	8.67 ± 0.61	-^b^	–	–
**2**	22.79 ± 0.71	58.56 ± 1.78	28.53 ± 1.47	–	28.58 ± 1.27
**3**	27.01 ± 0.82	31.83 ± 1.44	–	–	–
**4**	16.90 ± 1.22	24.72 ± 2.15	73.79 ± 1.70	–	–
**5**	5.84 ± 0.33	8.39 ± 0.89	4.05 ± 0.20	32.88 ± 1.85	–
**6**	5.99 ± 0.49	9.20 ± 1.27	15.86 ± 0.35	–	44.19 ± 1.25
**7**	9.84 ± 0.78	12.26 ± 0.64	13.34 ± 0.39	–	12.91 ± 0.30
**8**	9.12 ± 0.82	17.47 ± 1.77	10.72 ± 0.80	–	17.21 ± 0.41
**9**	24.05 ± 0.99	36.52 ± 2.56	9.04 ± 0.99	–	38.64 ± 1.22
**10**	5.43 ± 0.40	29.78 ± 2.31	40.10 ± 1.66	–	24.93 ± 0.82
**11**	10.59 ± 0.90	5.66 ± 0.28	65.77 ± 1.79	–	–
**12**	3.58 ± 0.11	4.25 ± 0.32	–	–	–
**13**	–	–	–	–	–
**14**	49.97 ± 1.78	3.60 ± 0.30	26.97 ± 1.01	–	–
**15**	13.06 ± 0.70	3.64 ± 0.22	–	–	–
**16**	21.14 ± 0.87	28.47 ± 1.78	45.56 ± 1.29	–	–
**17**	12.06 ± 1.19	10.55 ± 0.91	–	–	–
**18**	3.82 ± 0.31	5.24 ± 0.40	24.80 ± 1.58	–	–
**19**	20.13 ± 0.90	12.09 ± 1.52	–	–	–
**20**	10.43 ± 0.82	3.18 ± 0.20	20.04 ± 1.74	–	–
**21**	16.60 ± 0.91	23.14 ± 2.78	11.77 ± 0.60	30.76 ± 2.00	–
**22**	4.14 ± 0.21	6.54 ± 0.74	26.12 ± 0.73	–	–
**23**	25.31 ± 1.33	33.75 ± 2.89	–	–	–
**24**	24.49 ± 1.55	41.84 ± 2.97	52.75 ± 1.44	–	–
**25**	–	–	–	–	–
**26**	–	–	–	–	–
**27**	7.83 ± 0.40	39.80 ± 2.55	50.66 ± 1.82	–	–
**28**	10.15 ± 0.75	20.55 ± 2.11	16.70 ± 0.65	–	–
**29**	14.34 ± 0.91	16.92 ± 0.87	–	–	–
**30**	27.99 ± 1.31	25.09 ± 0.99	2.29 ± 0.17	–	–
**31**	3.49 ± 0.20	24.08 ± 1.18	33.48 ± 0.88	–	–
**32**	12.28 ± 0.41	16.15 ± 0.41	27.02 ± 0.90	–	–
**33**	4.96 ± 0.51	19.04 ± 0.65	26.20 ± 0.39	–	35.88 ± 0.79
**34**	5.37 ± 0.60	23.39 ± 0.88	22.99 ± 0.45	–	–
**35**	7.41 ± 0.77	7.41 ± 0.33	36.45 ± 0.70	–	–
**36**	4.01 ± 0.68	37.17 ± 1.40	28.98 ± 0.78	–	–
**37**	9.09 ± 0.81	21.48 ± 1.29	35.11 ± 0.49	–	–
**38**	40.81 ± 1.39	83.15 ± 2.91	–	–	–
**39**	–	–	–	–	–
**40**	6.73 ± 0.50	18.34 ± 0.80	35.61 ± 0.55	51.30 ± 2.55	–
**41**	14.55 ± 1.20	10.57 ± 0.46	28.80 ± 0.75	–	–
**42**	–	–	–	–	–
**43**	38.60 ± 0.61	43.99 ± 1.20	34.48 ± 0.77	24.75 ± 0.89	–
**44**	5.34 ± 0.30	–	9.24 ± 0.40	–	–
**45**	8.61 ± 0.28	–	15.59 ± 0.78	–	–
**46**	1.25 ± 0.07	–	5.37 ± 0.11	–	–
**47**	9.04 ± 0.29	10.25 ± 0.44	21.15 ± 0.49	–	–
**48**	27.41 ± 1.50	39.70 ± 1.39	50.96 ± 1.91	49.14 ± 1.39	–
**49**	32.76 ± 1.44	35.23 ± 1.40	48.49 ± 1.65	49.79 ± 0.47	–
**50**	20.69 ± 1.39	31.56 ± 1.20	29.09 ± 1.27	26.89 ± 0.56	–
**51**	42.04 ± 1.60	20.87 ± 1.09	31.25 ± 1.33	–	–
**52**	13.29 ± 0.87	15.29 ± 0.82	24.30 ± 1.19	–	–
**53**	17.49 ± 0.98	28.48 ± 1.14	57.32 ± 2.01	–	–
**54**	17.52 ± 1.52	28.51 ± 1.16	19.03 ± 0.71	–	–
**55**	5.87 ± 0.20	14.31 ± 1.07	34.10 ± 1.45	–	–
**56**	6.92 ± 0.44	12.16 ± 0.92	19.31 ± 0.82	34.46 ± 1.04	–
**57**	1.58 ± 0.06	3.22 ± 0.18	–	–	–
**58**	–	–	–	–	–
**59**	57.98 ± 1.71	–	–	–	–
**60**	10.70 ± 0.80	–	–	–	–
**61**	27.30 ± 1.00	26.87 ± 1.20	28.20 ± 1.49	–	27.06 ± 1.03
**62**	45.13 ± 1.40	86.18 ± 2.44	–	–	–
**63**	–	–	–	–	–
**64**	23.87 ± 1.37	38.29 ± 0.90	–	–	–
**65**	32.68 ± 1.22	30.53 ± 1.29	–	–	–
**66**	8.56 ± 0.49	30.29 ± 1.22	51.96 ± 1.70	–	19.59 ± 1.17
**67**	8.29 ± 0.42	12.07 ± 0.37	15.04 ± 0.60	50.26 ± 1.17	16.13 ± 0.82
**68**	19.65 ± 1.00	21.25 ± 0.80	–	–	37.41 ± 1.35
**69**	8.79 ± 0.61	7.95 ± 0.40	11.52 ± 0.39	36.82 ± 1.25	25.09 ± 0.54
**70**	3.09 ± 0.19	4.22 ± 0.20	3.00 ± 0.18	5.38 ± 0.30	3.15 ± 0.27
**71**	8.65 ± 0.33	8.09 ± 0.52	23.18 ± 0.78	–	20.80 ± 0.79
**72**	12.98 ± 0.77	22.55 ± 1.00	16.05 ± 0.91	–	44.91 ± 1.13
**73**	11.16 ± 0.81	25.58 ± 0.97	–	–	57.70 ± 1.44
**74**	26.79 ± 1.22	34.72 ± 1.20	–	31.83 ± 0.82	35.31 ± 1.25
**75**	9.27 ± 0.91	21.34 ± 1.04	32.07 ± 1.02	21.93 ± 0.61	–
**76**	18.00 ± 1.09	36.02 ± 1.20	–	50.20 ± 1.79	–
**77**	13.68 ± 1.29	19.44 ± 0.79	23.61 ± 1.45	–	51.81 ± 0.87
**78**	26.26 ± 2.35	35.22 ± 1.28	–	–	–
**79**	22.97 ± 1.75	–	–	–	–
**80**	–	–	–	26.81 ± 1.01	–
**81**	7.89 ± 0.56	27.77 ± 1.32	34.92 ± 1.70	–	–
**82**	24.16 ± 0.60	28.67 ± 1.21	8.95 ± 0.27	–	–
AMD	0.52 ± 0.14	0.81 ± 0.11	1.05 ± 0.09	0.92 ± 0.08	0.67 ± 0.08

a The standard deviation (SD) of three time independent tests.

b The standard deviation (SD) of three time independent tests.

Based on MDA-MB-231, the further structure–activity relationship was summarised as follows. First, about 2-phenylethenesulfonyl fluoride series, compounds with non-substituted or *para*-substituted of the benzene ring showed better activity (compounds **1, 5, 6, 11, 12, 14** and **15**); For the *meta*-substituted compounds, strong electron withdrawing group, such as **F, CF_3_, NO_2_** reflected great contribution to the activity (compounds **18, 20** and **22**). Two substitutions containing Cl were beneficial to the activity (compounds **69** and **71**). For the **R**-ethenesulfonyl fluoride, when **R** is heterocyclic or benzo heterocyclic, it was not beneficial to the activity (compounds **44, 45, 46, 58** and **59**). Second, for the series of diene-1-sulfonyl fluoride, most of these compounds had certain activity (compounds **51–56**), among them, compound 2-(fluorosulfonyl)vinyl)phenyl)buta-1,3-diene-1-sulfonyl fluoride showed the best activity with IC_50_ value of 3.22 µM.

It was obvious from the data that most compounds showed good activity against A375 cell (compounds **12, 18, 31, 46, 57** and **70**, their IC_50s_ value is around 3.0 µM), among them, compound **46** possessed the highest activity with the IC_50_ value of 1.25 µM, which is comparable with the positive control AMD. For the phenylethenesulfonyl fluoride series, most of the *para*-substituted derivatives showed good activity except for –CN and –COOEt substitutions. But, in addition to F substitution, most of the *meta*-substituted derivatives displayed weak activity. We then investigated the SAR profiles of the multi-substituted group of ethenesulfonyl fluoride, compared to phenylethenesulfonyl fluoride moiety, the kind of diene-1-sulfonyl fluoride moiety could not significantly improve the activity.

### 3.3. Inhibition assay of human normal cell

In order to determine the selective cancer cell toxicity of some title compounds. We subsequently conducted a proliferative inhibition assay with human normal liver cell (L-02) and gastric mucosa cell (GES-1). As shown in Table 2, all compounds manifested obvious un-toxic effect on GES-1 and L-02 with IC_50_ from 1.33 to 2.80 mM. The data indicated that the title compounds have good selectivity against tumour cells over somatic cells (Tables 1 and 2).

**Table 2.** Selected compounds against normal cells L-02 and GES-1proliferation.^a^

Compound	L-02 (IC_50_, mM)	GES-1 (IC_50_, mM)
**1**	1.97 ± 0.20	1.45 ± 0.17
**5**	1.33 ± 0.15	2.00 ± 0.25
**15**	2.11 ± 0.19	2.50 ± 0.22
**25**	2.45 ± 0.25	2.80 ± 0.31
**31**	1.41 ± 0.10	1.65 ± 0.18
**46**	2.06 ± 0.18	1.82 ± 0.11
**57**	2.01 ± 0.16	2.19 ± 0.18
**75**	2.44 ± 0.22	1.89 ± 0.17

a MTT assays were used for evaluation, and values were expressed as mean IC_50_ of the triplicate experiment.

### 3.4. Telomerase activity

Screening results of cell activity showed that most compounds had good activity against A375 cell, due to high expression of telomerase in melanoma cells A375, so, to confirm if the title compounds performed anticancer activity via telomerase inhibition, some selected compounds were evaluated using an extraction from A375 cells. The results are summarised in Table 3. Among them, compounds **5**, **31**, **36**, **57**, **46** and **70** showed potent inhibitory activities against telomerase with IC_50_ values less than 1.5 µM, better than positive control staurosporine with IC_50_ value 8.67 µM. One of them, compound **5** showed the most potent activity against telomerase with IC_50_ value of 0.71 µM, which is 12 times as much as the positive control staurosporine.

**Table 3.** Some compounds inhibitory activity against telomerase.^a^

Compound	IC_50_ (µM)	Compound	IC_50_ (µM)
**1**	2.41 ± 0.51	**46**	1.12 ± 0.27
**5**	0.71 ± 0.28	**47**	2.13 ± 0.60
**6**	2.98 ± 0.59	**48**	40.15 ± 1.30
**7**	3.02 ± 0.44	**55**	47.22 ± 1.52
**8**	4.33 ± 0.77	**56**	3.20 ± 0.55
		**57**	0.97 ± 0.12
**12**	2.77 ± 0.58	**58**	39.88 ± 1.29
**13**	38.99 ± 1.29	**59**	44.21 ± 2.26
**15**	1.87 ± 0.41	**62**	39.89 ± 1.40
**17**	10.08 ± 1.13	**63**	45.33 ± 1.87
**18**	1.09 ± 0.11	**67**	1.87 ± 0.47
**20**	4.17 ± 0.39	**69**	4.22 ± 0.71
**22**	2.25 ± 0.30	**70**	1.01 ± 0.13
**25**	no	**71**	6.08 ± 1.30
**31**	0.81 ± 0.07	**75**	4.40 ± 2.00
**36**	1.44 ± 0.22	**80**	49.27 ± 2.55
**39**	48.76 ± 2.22	**81**	2.55 ± 0.40
**42**	no	**82**	37.88 ± 2.19
**44**	15.22 ± 1.56		
Staurosporine^b^	8.67 ± 0.18	Staurosporine^b^	8.67 ± 0.18

^a^Telomerase supercoiling activity.

^b^Staurosporine is reported as a control.

From the structure–activity relationships presented in Table 3, it can be concluded that most compounds had high telomerase activity. Overall, the compounds with low activity against cancer cell expressed the same weak telomerase activity (compounds **25** and **42**). There was also a good correlation between the antiproliferative activity and the IC_50s_ of telomerase inhibition activity (compounds **31, 47, 57** and **70**).

In order to further analyse the SAR, the 82 compounds were divided into two types, seriers one is **R**-ethenesulfonyl fluoride skeleton and series two is **R^1^**-diene-1-sulfonyl fluoride skeleton. In general, the activity of series one is generally higher than the activity of series two (compounds **5, 15, 18, 31, 36, 46, 47, 58, 67** with **48** and **55**). Further SAR analysis was focused on different substitutes of **R**. For the series one skeleton, when **R** was a different substitute phenyl、thick ring and heterocyclic ring showed significant effect on the activity against telomerase (compounds **5**, **31**, **46**, **47** and **58**). For series two skeleton, the nucleus of different rings had little effect on the activity of telomerase (compounds **48** and **55**). The result of enzyme inhibition test showed that title compounds had moderate activity against telomerase. For this reason, it is not certain that TERT is the only protein target, responsible for the biological activity.

## 4. Conclusions

Inspired by the dual warhead approach, based on our previous TERT model of rational drug design, in this study, 82 sulfonyl fluoride derivatives were designed, synthesised and biologically evaluated as potential telomerase inhibitors. Total 82 compounds were evaluated for their antiproliferative activities against A375, MDA-MB-231, SMMC-7721, SGC-7901 and MGC-803 cell lines. The structure–activity relationships were discussed in depth. The results showed most compounds had good activity against A375 cell with lower toxicity against normal cells *in vitro*. Moreover, compounds **5, 31** and **57** displayed high inhibitory activity against telomerase with IC_50_ = 0.71, 0.81 and 0.97 µM, respectively. These results are of help in rational design of more efficient telomerase TERT inhibitors in the future.
